# Primary analysis of a prospective, randomized, single-blinded phase II trial evaluating the HER2 peptide GP2 vaccine in breast cancer patients to prevent recurrence

**DOI:** 10.18632/oncotarget.11751

**Published:** 2016-08-31

**Authors:** Elizabeth A. Mittendorf, Alexandros Ardavanis, Jennifer K. Litton, Nathan M. Shumway, Diane F. Hale, James L. Murray, Sonia A. Perez, Sathibalan Ponniah, Constantin N. Baxevanis, Michael Papamichail, George E. Peoples

**Affiliations:** ^1^ Department of Breast Surgical Oncology, The University of Texas MD Anderson Cancer Center, Houston, TX, USA; ^2^ Cancer Immunology and Immunotherapy Center, St. Savas Cancer Hospital, Athens, Greece; ^3^ Department of Breast Medical Oncology, The University of Texas MD Anderson Cancer Center, Houston, TX, USA; ^4^ Department of Hematology/Oncology, Brooke Army Medical Center, Ft. Sam Houston, TX, USA; ^5^ Department of Surgery, Brooke Army Medical Center, Ft. Sam Houston, TX, USA; ^6^ Cancer Vaccine Development Laboratory, Department of Surgery, Uniformed Services University of the Health Sciences, Bethesda, MD, USA; ^7^ Cancer Vaccine Development Program, San Antonio, TX, USA

**Keywords:** vaccine, cytotoxic T lymphocytes, breast cancer, HER2, trastuzumab

## Abstract

GP2 is a HER2-derived, HLA-A2+ restricted peptide. Phase I studies showed GP2 administered with GM-CSF to be safe and immunogenic. Here we report the primary analysis of a prospective, randomized, multicenter phase II adjuvant trial conducted to determine the vaccine's efficacy. The trial enrolled HLA-A2+, clinically disease-free, node-positive and high-risk node-negative breast cancer patients with tumors expressing HER2 (immunohistochemistry[IHC] 1+-3+). Patients were randomized to GP2+GM-CSF versus GM-CSF alone. Disease-free survival (DFS) was analyzed in intention-to-treat (ITT) and per-treatment cohorts; pre-specified subgroup analyses were performed for patients with IHC 3+ or FISH+ disease. The trial enrolled 180 patients; 89 received GP2+GM-CSF and 91 received GM-CSF alone. The groups were well-matched for clinicopathologic characteristics. Toxicities have been minimal. The Kaplan-Meier estimated 5-year DFS rate in the ITT analyses was 88% (95% CI:78-94%) in vaccinated *vs*. 81% (95% CI:69-89%) (*P* = 0.43) in control patients after a 34 month median follow-up. In the per-treatment analysis, the estimated 5-year DFS rates were 94% (95% CI:83-98%) and 85% (73-92%) (*P* = 0.17). In IHC 3+/FISH+ patients, the estimated 5-year DFS rate was 94% (82-98%) in vaccinated patients (*n* = 51) *vs*. 89% (71-96%) in control patients (*n* = 50), (*P* = 0.86) in the ITT analyses and 100% *vs*. 89% (71-96%) in vaccinated *vs*. control patients in the per-treatment analyses (*P* = 0.08). While the overall ITT analysis did not demonstrate benefit to vaccination, this trial confirmed that the GP2 vaccine is safe and suggests that vaccination may have clinical activity, particularly in patients with HER2 overexpression who received the full vaccine series (ie per-treatment group).

## INTRODUCTION

Vaccines targeting tumor-associated antigens (TAA) are a form of immunotherapy that could be used in treating cancer patients. In breast cancer, the most studied TAA is HER2, and several peptides derived from the HER2 protein have been shown to elicit immune responses. Our group has been investigating HER2-derived, major histocompatibility class (MHC) class I peptides combined with the immunoadjuvant granulocyte-macrophage colony-stimulating factor (GM-CSF) as a vaccine strategy to stimulate a CD8^+^ T cell response. One such peptide is E75 (nelipepimut-S) which, in a phase I/II clinical trial vaccinating breast cancer patients in the adjuvant setting to prevent disease recurrence, was found to have a five year disease free survival (DFS) rate of 90% in vaccinated patients versus 80% in unvaccinated control patients [1]. A second is GP2 (aa:654-662:IISAVVGIL). GP2 has a lower HLA-A2 binding affinity than nelipepimut-S; therefore, is a subdominant epitope [2, 3]. Kuhns et al. used crystallography to show that the poor binding of GP2 to MHC class I molecules exists in part because the center of the peptide does not make stabilizing contact with the MHC class I molecule binding cleft [4]. However, this flexibility of the center of the peptide may improve the peptide's immunogenicity because it can assume multiple different conformations thereby stimulating multiple T cell populations with different T-cell receptors (TCR). Despite the lower binding affinity, GP2 has been shown to induce a CD8^+^ T cell response that is of similar magnitude to nelipepimut-S [5, 6]. Furthermore, in previous trials evaluating nelipepimut-S+GM-CSF, we have seen evidence of intra-antigenic epitope spreading with clonal expansion of GP2-specific CD8^+^ T cells. Taken together, these data provide evidence of the immunogenicity of GP2.

Our group conducted the first in-human phase I trial of GP2+GM-CSF in breast cancer patients [7]. The trial showed that the vaccine is safe with minimal local and systemic toxicity. In addition, vaccination elicited antigen specific immune responses against both the immunizing peptide (GP2) and E75 demonstrating epitope spreading. Subsequently, we have conducted a prospective, randomized, multi-center phase II trial investigating GP2+GM-CSF administered in the adjuvant setting to node-positive and high-risk node-negative breast cancer patients with tumors expressing any degree of HER2 (immunohistochemistry [IHC] 1-3+) (NCT00524277). The trial enrolled HLA-A2+ patients that were randomized to receive GP2+GM-CSF versus GM-CSF alone. The trial's primary objective was to determine if vaccination with GP2+GM-CSF versus GM-CSF alone could reduce the recurrence rate. Here, we report the trial's primary efficacy analysis, which, per the statistical analysis plan, was conducted after 39 DFS events occurred.

## RESULTS

### Patients

**Figure 1:**
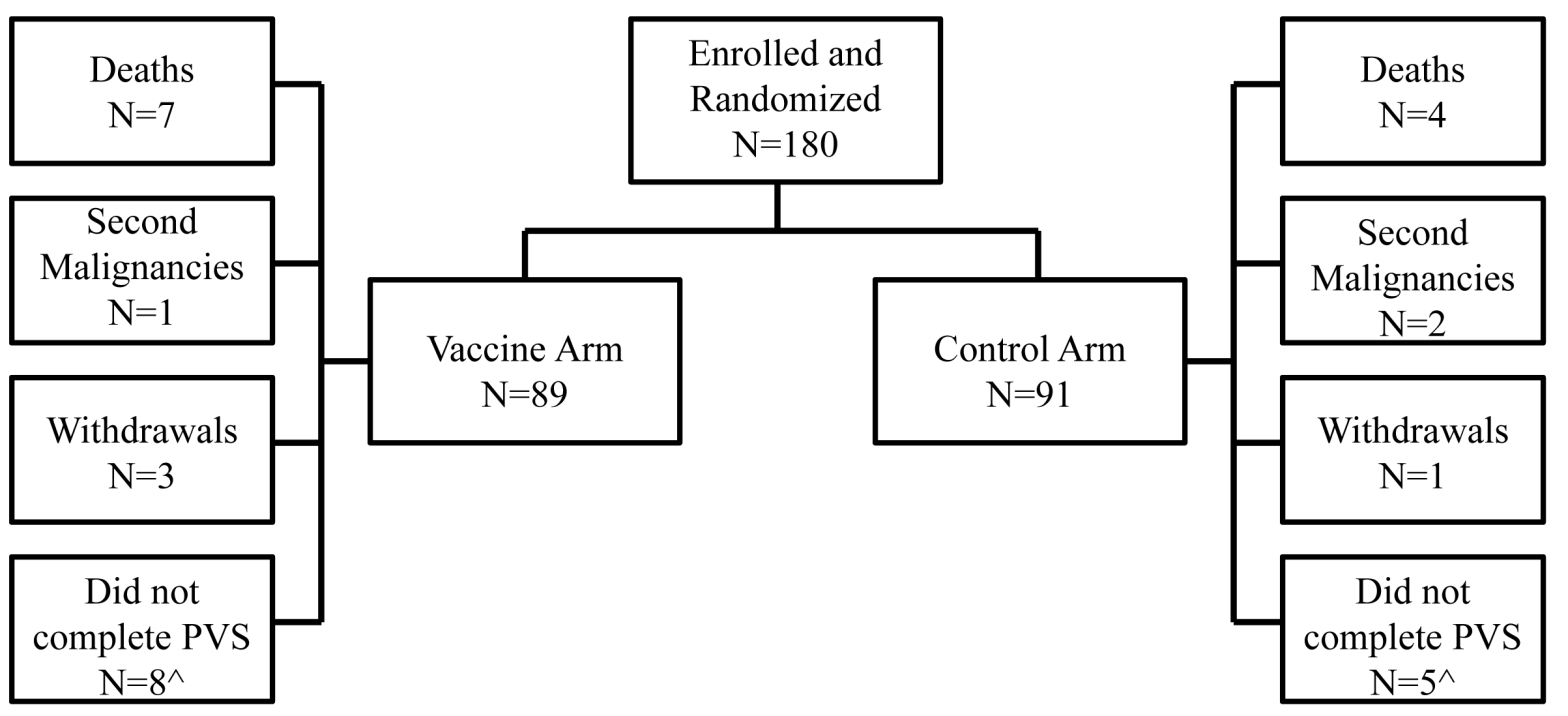
Consort diagram Flow of patients through the study. ^The number of patients that did not complete the primary vaccination series (PVS) includes patients that withdrew, met the primary endpoint (recurrence, second malignancy, or death from any cause), or chose not to continue on study before completing the PVS.

The trial enrolled 180 HLA-A2+ patients; 89 were randomized to the GP2+GM-CSF vaccine group and 91 to the GM-CSF only control arm (Figure 1). There were no differences with respect to clinicopathologic characteristics between groups (Table 1).

**Table 1:** Clinicopathologic characteristics by treatment group

Characteristic	No. (%) of Vaccinated Patients (*N* = 89)	No. (%) of Controls (*N* = 91)	*P* value^^^
Median age (years) (range)	49 (27-77)	50 (26-72)	0.98
T stage			0.46
T0/is	2 (2%)	3 (3%)	
T1	36 (40%)	38 (42%)	
T2	39 (44%)	34 (37%)	
T3	6 (7%)	12 (13%)	
T4	4 (5%)	4 (4%)	
Tx	2 (2%)	0 (0%)	
Nodal status			0.30
Positive	51 (57%)	60 (66%)	
Negative	38 (43%)	31 (34%)	
Not done	0 (0%)	1 (1%)	
Histology			0.31
Ductal	86 (96%)	83 (91%)	
Lobular	2 (3%)	5 (6%)	
Other	1 (1%)	3 (3%)	
Grade			1.00
Moderate/well differentiated	38 (43%)	39 (43%)	
Poorly differentiated	51 (57%)	52 (57%)	
ER/PR status			0.64
Positive	55 (62%)	60 (66%)	
Negative	34 (38%)	31 (34%)	
HER2 status			0.77
Positive	51 (57%)	50 (55%)	
Negative	38 (43%)	41 (45%)	
Surgery			0.50
Lumpectomy	36 (40%)	32 (35%)	
Mastectomy	53 (60%)	57 (63%)	
Other	0 (0%)	1 (1%)	
Unknown	0 (0%)	1 (1%)	
Post-mastectomy radiation^*^			0.31
Yes	34 (64%)	42 (74%)	
No	19 (36%)	15 (26%)	
Chemotherapy			0.56
Yes	82 (92%)	86 (95%)	
No	7 (8%)	5 (5%)	
Endocrine therapy in hormone receptor–positive patients			0.62
Yes	54 (98%)	57 (95%)	
No	1 (2%)	3 (5%)	
Trastuzumab in HER2-positive patients			0.36
Yes	47 (92%)	43 (86%)	
No	4 (8%)	7 (14%)	

^ Unknown values were not included in statistical analyses

* Data regarding postmastectomy radiation only includes patients undergoing mastectomy

### Toxicity

**Figure 2:**
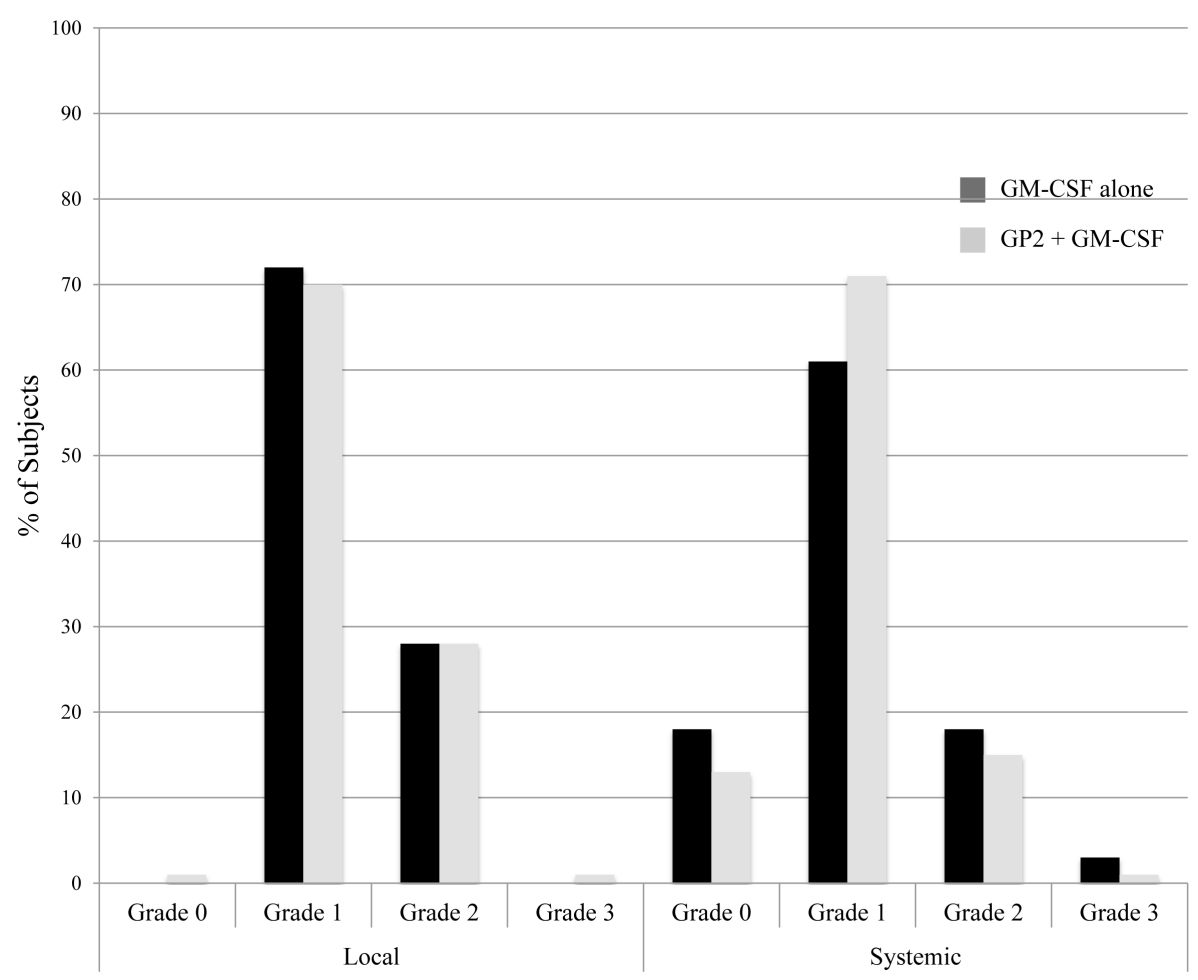
Maximum toxicity The maximum local and systemic toxicity experienced by patients administered the GP2+GM-CSF vaccine were comparable to those experienced by patients receiving GM-CSF alone.

For patients receiving GP2+ GM-CSF, maximum local toxicities experienced during the primary vaccination series (PVS) were grade 1(70%), grade 2(28%), or grade 3 (1%) (Figure 2). The most common toxicities included erythema, induration and pruritis; the grade 3 toxicity was induration. Maximum systemic toxicities were grade 0(13%), grade 1(71%), grade 2(15%), or grade 3(1%). The most common systemic toxicities included fatigue, headache, and myalgias; the grade 3 toxicity was a diffuse maculopapular rash. The toxicities were comparable for patients receiving GM-CSF only, with maximum local toxicities being grade 1(75%) or grade 2(25%); and maximum systemic toxicities being grade 0(21%), grade 1(60%), grade 2(15%), or grade 3 (3%). The grade 3 systemic toxicities in this group included diffuse urticarial reactions, syncope and extremity pain.

### Immunologic response

**Figure 3:**
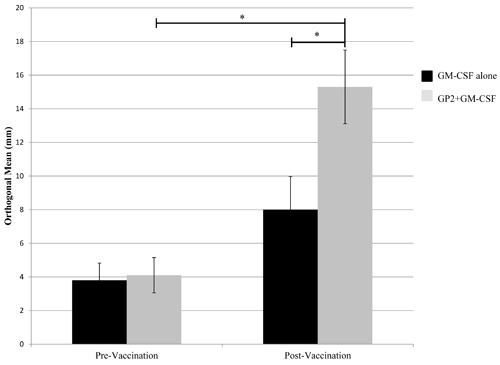
*In vivo* immunologic response to vaccination *In vivo* immune responses were determined using a delayed type hypersensitivity (DTH) reaction. Patients who were vaccinated with GP2+GM-CSF had a significant increase in their DTH reaction to both the immunizing peptide post-vaccination compared to pre-vaccination. (* = *P* < 0.001).

*In vivo* immunologic responses were evaluated using a delayed type hypersensitivity (DTH) reaction. For vaccinated patients, there was a significant increases in post-vaccination DTH reactions compared to pre-vaccination DTH reactions (Figure 3). In vaccinated patients, the average (± standard error) orthogonal mean to GP2 prior to vaccination was 4.1±1.1mm versus 15.3± 2.2mm post-vaccination (*P* < 0.001). In addition, the post-vaccination response was significantly greater in vaccinated patients than in control patients (15.3± 2.2mm *vs*. 8.0±2.0mm, *P* < 0.001). For patients receiving GM-CSF alone, the average orthogonal mean prior to inoculation was 3.9±1.0mm versus 8.0±2.0mm post-vaccination (*P* = 0.12).

**Figure 4:**
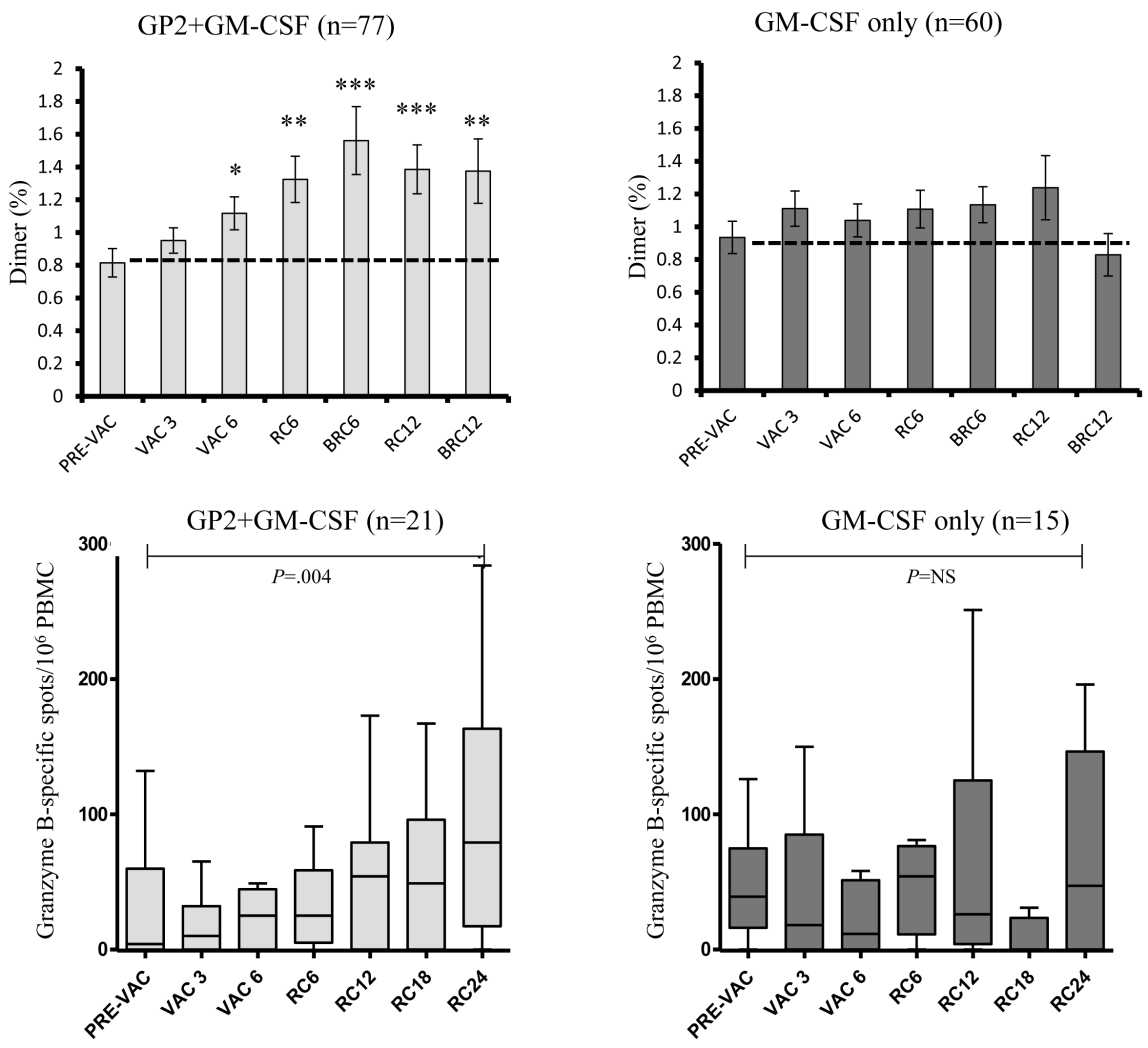
*Ex vivo* immunologic response to vaccination *Ex vivo* immune responses were determined for GP2-specific CTL clonal expansion by Ig:A2 dimer assays and CTL function by granzyme B ELISPOT. The GP2+GM-CSF vaccine induced significant increases in both clonal expansion as well as improved CTL function compared to pre-vaccine levels while GM-CSF alone had no such affect. (RC6 = response 6 months after primary vaccination series [PVS] completion, BRC6 = response 1 month after booster #1 was administered [occurred 6 months after PVS completion], RC12 = response 12 months after PVS completion, BRC12 = response 1 month after booster #2 was administered [occurred 12 months after PVS completion], RC18 = response 18 months after PVS, RC24 = response 24 months after PVS; * = *P* < 0.05, ** = *P* < 0.01, *** = *P* < 0.001).

Additionally, *ex vivo* immune responses were assessed by phenotypic clonal expansion assays in the majority of patients (*n* = 137) and by T cell functional assays in a consecutive subset of patients (*n* = 36) (all patients enrolled at a single site) (Figure 4). GP2-specific CTL were quantified using the dimer assay and demonstrated a gradual expansion over time reaching statistical significance post-vaccination compared to baseline in the vaccine patients (*N* = 77) but not in the control patients (*n* = 60). At a single site, all consecutive patients were assessed for CTL functionality by granzyme B secretion and demonstrated a significant increase compared to baseline in the vaccinated patients (*n* = 21) but not in the control patients (*n* = 15).

### Disease-free survival

**Figure 5:**
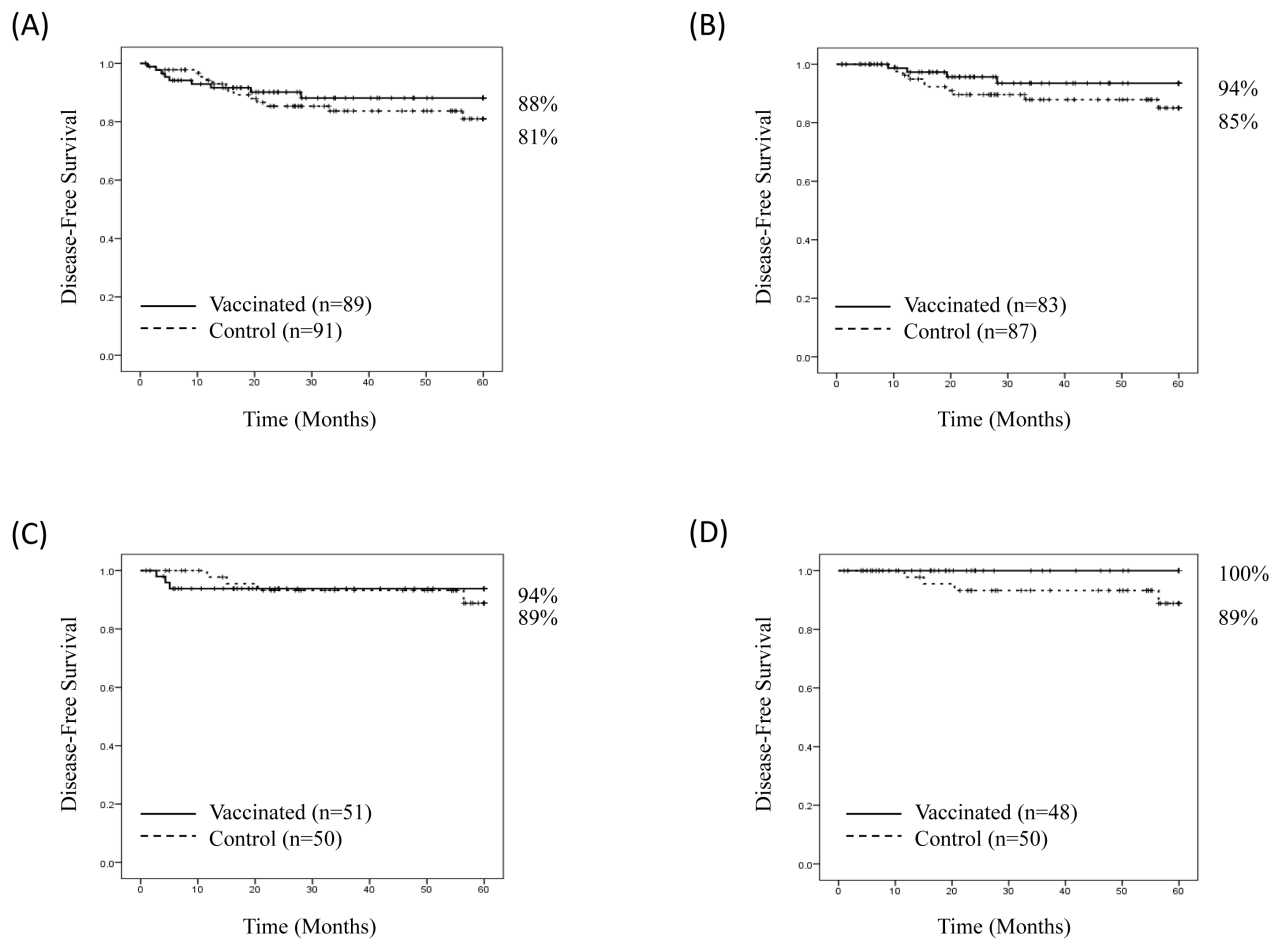
Disease-free survival Disease-free survival is shown for **A.** all patients, intention to treat, **B.** all patients, per treatment, **C.** patients with HER2-positive breast cancer, intention to treat, and **D.** patients with HER2-positive breast cancer, per treatment.

At the time of the primary analysis, the median follow-up was 34 months (range 1-60 months). For the intention-to-treat (ITT) population, the Kaplan-Meier estimated 5-year DFS rate was 88% (78-94%) in vaccinated patients versus 81% (69-89%) in control patients (*P* = 0.43) (Figure 5A). There were 10 patients excluded from the per-treatment analysis; six from the vaccinated group (two second malignancies and four recurrences during the PVS) and four from the control group (two second malignancies and two recurrences during the PVS). The estimated 5-year DFS rate in the per-treatment analysis was 94% (83-98%) in vaccinated patients (*n* = 83) versus 85% (73-92%) in control patients (*n* = 87) (*P* = 0.17) (Figure 5B). In the ITT, planned subset analysis of patients with HER2+ disease, the estimated 5-year DFS rate was 94% (82-98%) in vaccinated patients (*n* = 51) versus 89% (71-96%) in control patients (*n* = 50) (*P* = 0.86). (Figure 5C). Three vaccinated patients recurred during the PVS, and therefore were excluded from the per-treatment analysis. None of the control patients recurred during the PVS. The estimated 5-year DFS rate in the per-treatment analysis was 100% in vaccinated patients (*n* = 48) *vs*. 89% (71-96%) in control patients (*n* = 50), (*P* = 0.08) (Figure 5D).

## DISCUSSION

Here, we report the primary analysis of the phase II trial evaluating GP2+GM-CSF administered in the adjuvant setting to disease-free, node-positive and high-risk node-negative breast cancer patients to prevent recurrence. The ITT analysis of the entire randomized population did not show a statistically significant reduction in the recurrence rate in vaccinated patients therefore this was a negative study. However, the study was highly suggestive that the vaccine may be effective in select patients, specifically those with HER2-positive tumors who also receive trastuzumab, consistent with preclinical studies demonstrating synergy between these two forms of immunotherapy.

Our group has previously conducted a phase I/II clinical trial vaccinating node-positive and high-risk node-negative breast cancer patients with nelipepimut-S and GM-CSF in the adjuvant setting [1]. That trial enrolled 187 evaluable patients including 108 that were vaccinated and 79 unvaccinated controls. In the final analysis conducted after 60 months of follow-up, the 5-year DFS rate was 90% in the vaccinated group versus 80% in the control group (*P* = 0.08), a 48% reduction in relative recurrence risk. Consistent with this, and confirming the potential efficacy of a single MHC-class I epitope vaccine, the current trial evaluating GP2+GM-CSF demonstrated a 36% reduction in relative recurrence risk in the ITT population and a 60% reduction in relative risk in the per treatment population. The trial evaluating nelipepimut-S had several limitations. In that study, patients had their HLA type determined and HLA-A2 or HLA-A3+ patients were vaccinated. HLA-A2 and HLA-A3- patients served as unvaccinated controls. The trial was therefore not blinded, and there was no group that received GM-CSF alone. The current study investigating GP2 addressed several of those limitations. Specifically, the study only enrolled HLA-A2+ patients who were randomized to GP2+GM-CSF versus GM-CSF alone. In addition, the patients were blinded to their treatment arm.

The current study also confirms the finding from the phase I trial evaluating GP2+GM-CSF that the vaccine is safe and well-tolerated [7]. The majority of patients experienced only grade 1 local and systemic toxicities. Importantly, toxicities in the GM-CSF only group were comparable to those seen in the vaccinated group, suggesting the toxicities are attributable to the GM-CSF immunoadjuvant. This is consistent with data showing a similar toxicity profile in a phase II trial evaluating the AE37+GM-CSF vaccine administered in the same patient population [8]. Although the toxicities can be attributed to the GM-CSF immunoadjuvant, the immune responses cannot. This is evidenced by the *in vivo* and *ex vivo* immune monitoring data. Comparing the pre- to post-treatment DTH response, patients vaccinated with GP2+GM-CSF experienced a significant increase in their DTH response while those receiving GM-CSF only did not. Likewise, both CTL clonal expansion and enhanced CTL function were induced by the vaccine but not by GM-CSF alone. As discussed above, the early phase nelipepimut-S + GM-CSF trials did not have a GM-CSF alone control arm; therefore, the observation in the current trial that the GM-CSF is responsible for the toxicity associated with these peptide vaccines but not the immunologic response was previously unknown.

The current analysis suggests possible clinical benefit in a particular patient population. Specifically, in the per-treatment analysis in HER2-positive patients, there were no recurrences in patients who were vaccinated following completion of standard of care therapy that included trastuzumab. This is consistent with an observation that we had previously made in the early phase nelipepimut-S + GM-CSF trial. While enrollment to that study largely predated the routine use of trastuzumab in the adjuvant setting for HER2-positive breast cancer patients, a small number (*n* = 12) of HER2-positive patients were vaccinated after completion of an adjuvant systemic therapy regimen that included trastuzumab [9]. In those patients, after 5 years of follow-up, there were no recurrences (unpublished data). Taken together, the findings from these two trials suggest synergy between trastuzumab and HER2-derived, MHC class I CD8+ T cell-eliciting vaccines.

In a series of preclinical studies using a HER2/*neu* transgenic mouse model, Jaffee and colleagues showed that both cellular and humoral anti-neu immune responses are required to eradicate HER2/*neu* expressing tumors. In an initial study, they showed that neu-specific vaccination resulted in the generation of neu-specific CTL but little neu-specific IgG. In contrast, vaccination of parental FVB/N mice that are non-tolerogenic resulted in significant induction of both neu-specific CTL and neu-specific IgG, and these mice were fully protected from tumor challenge [10]. *In vivo* lymphocyte and NK cell depletion studies performed in the FVB/N mice confirmed that both cellular and humoral neu-specific responses were required for tumor eradication [11]. Subsequent to that, they showed that HER2/*neu* transgenic mice treated sequentially with neu-specific monoclonal antibodies and a neu-targeted GM-CSF secreting whole cell vaccine could overcome immune tolerance leading to improvements in tumor-free survival over either modality alone [12]. *In vivo* lymphocyte depletion studies confirmed that the antitumor effects of either the antibodies, vaccine or the combination was dependent on CD4+ and CD8+ T cells [12].

There is also clinical evidence suggesting an important role for lymphocytes in mediating trastuzumab response. First, the clinical response seen in trastuzumab-treated patients occurs over a week after initiation of therapy which is consistent with a T-lymphocyte-mediated lytic effect based in part on the timing of tumor antigen transport to draining lymph nodes after *in vivo* tumor cell lysis [13, 14]. Second, in a study of patients with HER2-positive breast cancer receiving trastuzumab, Taylor et al. demonstrated generation of a HER2-specific CD4^+^ T cell response in 6 of 10 evaluable patients [15]. In that same study, investigators showed anti-HER2 antibodies in approximately 60% of 27 evaluable patients during treatment and found that these anti-HER2 humoral responses significantly increased during therapy and were associated with improvements in clinical response [15]. Knutson et al. showed that patients treated on the trastuzumab arm of the NCCTG 9831 adjuvant therapy trial developed HER2-specific antibody responses [16]. Cox modeling suggested that larger increases in antibody responses were associated with improved DFS (HR = 0.23, *P* = .04). Taken together, these preclinical and clinical data suggest that induction of a broad antigen-specific immune response is important, providing strong rationale for combination of trastuzumab with vaccination.

In the current study, patients were not vaccinated until after completion of standard of care therapy which, in the case of HER2-positive patients, meant completion of trastuzumab. One important observation is that among HER2-positive patients, there were three patients who recurred during the PVS. Per protocol, these patients were excluded from the per treatment analysis. However, in light of the compelling evidence regarding potential synergy, this has led us to question whether the early recurrences may have been prevented with concurrent vaccination and trastuzumab. In a study of 21 patients with stage IV HER2-positive breast cancer being treated with trastuzumab, Disis et al. showed that concurrent vaccination with a vaccine designed to elicit HER2-specific T-helper immunity was safe [17]. Our group has completed a phase I study of the concurrent administration of trastuzumab and GP2+GM-CSF in early stage HER2-positive breast cancer patients. The study enrolled 17 patients. There was no dose-limiting or grade 3-5 local or systemic toxicities to include cardiac toxicity [18]. Based on these data, we would propose that further development of GP2 should include a phase II study investigating the vaccine administered in combination with trastuzumab. A similar study is currently ongoing which is enrolling patients with high-risk HER2-positive breast cancer in the adjuvant setting and randomizing them to trastuzumab versus trastuzumab plus nelipepimut-S + GM-CSF (NCT02297698).

## MATERIALS AND METHODS

### Patient characteristics and clinical protocols

The study was a prospective, randomized, single-blinded, multi-center trial (NCT00524277) conducted under an investigational new drug application (BB-IND#11730). The Institutional Review Board at all enrolling sites approved the study. To be eligible, patients had to have node-positive or high-risk node-negative breast cancer expressing some degree of HER2 (IHC 1-3+ and/or fluorescence in situ hybridization [FISH] ratio > 1.2). High-risk node-negative was defined as tumors ≥T2, grade 3, hormone receptor (HR)-negative, HER2 3+ by IHC or FISH > 2.0 (prior to CAP/ASCO guideline changes), had lymphovascular invasion, or had isolated tumor cells (N0(i+)) in the sentinel lymph node(s). Enrollment occurred 1-6 months after completion of standard therapy with surgery, chemotherapy, and if indicated, radiation. Patients receiving endocrine therapy continued on their prescribed regimen. Because GP2 is HLA-A2 restricted, patients had their HLA-A2 status determined, and HLA-A2+ were randomized to GP2+GM-CSF or GM-CSF alone. The trial's objective was to determine if there are differences in the recurrence rate for patients administered GP2+GM-CSF versus those receiving GM-CSF alone.

### Vaccine and vaccination series

The GP2 peptide was produced in good manufacturing practices grade and purified to >95%. Sterility, endotoxin, and general safety testing were carried out by the manufacturer with purity/stability testing every two years. Lyophilized peptide was reconstituted in 0.5mL of sterile saline at a dose of 500μg GP2. The peptide was mixed with 125μg GM-CSF and sterile saline was added to a final 1ml volume. For immunoadjuvant only patients, 125μg GM-CSF was diluted to a final 1ml volume with sterile saline. The PVS consisted of six inoculations given every 21-28 days. The 1.0ml inoculation (vaccine or GM-CSF alone) was split, with 0.5 ml given intradermally at two sites, 5cm apart in the same lymph node draining area (upper thigh). Once data from the phase I/II nelipepimut-S trial suggested that booster inoculations helped maintain peptide-specific immunity, a booster series was incorporated into this trial [19]. Four booster inoculations were administered in the same extremity as the primary series at 12, 18, 24 and 30 months from the enrollment date.

### Immune monitoring

A DTH reaction was used to assess *in vivo* immune responses. Briefly, 100μg of GP2 in 0.5mL normal saline and 0.5mL normal saline (volume control) were injected intradermally pre-vaccination and 1 month after completion of the PVS. Forty-eight to seventy-two hours after injection, the DTH was measured in two dimensions using the sensitive ballpoint-pen method [20]. Data were recorded as the orthogonal mean. A DTH response was determined in all patients regardless of randomization.

Additionally, *ex vivo* immune responses were assessed by phenotypic clonal expansion assays in the majority of patients (*n* = 137) and by T cell functional assays in a consecutive subset of patients (*n* = 36) (all patients enrolled at a single site). GP2-specific CTL were quantified using the Ig:A2 dimer molecule (BD) holding the GP2 peptide and analyzed by flow cytometry as previously described (reference) [7]. Functionally, granzyme B was assessed by standard ELISPOT as previously described [8].

### Clinical recurrences of disease

Patients were evaluated for disease recurrence per standard screening dictated by their treating physicians. Patients were considered to have recurrent disease if the recurrence was biopsy-proven or if they were treated for recurrence.

### Statistical analysis

The trial was designed as an exploratory study with 80% power to detect a 0.45 hazard ratio with a one-sided alpha of 0.10. The statistical analysis plan called for this primary analysis to be performed after 39 events occurred. Clinico-pathologic data were compared between groups. Median and range were used to summarize age, and the groups were compared using analysis of variance techniques. Baseline categorical variables were summarized with frequencies and proportions, and groups were compared using Fisher's exact test or Chi-Square test. DTH data are presented as means ± standard errors and compared using a Student *t* test. DFS was calculated from the date of randomization to the date of recurrence or death due to any cause, and otherwise was censored at date of last contact. DFS was analyzed using Kaplan-Meier techniques, and groups were compared using the log-rank test. Both an ITT (all randomized patients) and a per treatment analysis (all randomized patients minus those who recurred during the PVS (and therefore could not complete the PVS) or developed a second malignancy) were specified. A pre-specified analysis of patients with HER2-positive disease, defined as HER2 3+ by IHC or FISH > 2.0, was also performed.
